# The contribution of gamma bursting to spontaneous gamma activity in schizophrenia

**DOI:** 10.3389/fnhum.2023.1130897

**Published:** 2023-05-03

**Authors:** Kevin M. Spencer, Alexander Nakhnikian, Yoji Hirano, Margaret Levin

**Affiliations:** ^1^Research Service, VA Boston Healthcare System, Department of Psychiatry, Harvard Medical School, Boston, MA, United States; ^2^Department of Psychiatry, Faculty of Medicine, University of Miyazaki, Miyazaki, Japan; ^3^Department of Neuropsychiatry, Graduate School of Medical Sciences, Kyushu University, Fukuoka, Japan; ^4^EPhysBio LLC, Kingston, RI, United States

**Keywords:** schizophrenia, EEG, auditory steady-state response, gamma, gamma burst, spectral slope

## Abstract

Increased spontaneous gamma (30–100 Hz) activity (SGA) has been reported in the auditory cortex in schizophrenia. This phenomenon has been correlated with psychotic symptoms such as auditory hallucinations and could reflect the dysfunction of NMDA receptors on parvalbumin-expressing inhibitory interneurons. Previous findings are from time-averaged spectra, so it is unknown whether increased spontaneous gamma occurs at a constant level, or rather in bursts. To better understand the dynamical nature of spontaneous gamma activity in schizophrenia, here we examined the contribution of gamma bursting and the slope of the EEG spectrum to this phenomenon. The main results from this data set were previously reported. Participants were 24 healthy control participants (HC) and 24 matched participants with schizophrenia (SZ). The data were from EEG recordings during auditory steady-state stimulation, which were localized to bilateral pairs of dipoles in auditory cortex. Time-frequency analysis was performed using Morlet wavelets. Oscillation bursts in the gamma range were defined as periods during which power exceeded 2 standard deviations above the trial-wide average value for at least one cycle. We extracted the burst parameters power, count, and area, as well as non-burst trial power and spectral slope. Gamma burst power and non-burst trial power were greater in SZ than HC, but burst count and area did not differ. Spectral slope was less negative in SZ than HC. Regression modeling found that gamma burst power alone best predicted SGA for both HC and SZ (> = 90% of variance), while spectral slope made a small contribution and non-burst trial power did not influence SGA. Increased SGA in the auditory cortex in schizophrenia is accounted for by increased power within gamma bursts, rather than a tonic increase in gamma-range activity, or a shift in spectral slope. Further research will be necessary to determine if these measures reflect different network mechanisms. We propose that increased gamma burst power is the main component of increased SGA in SZ and could reflect abnormally increased plasticity in cortical circuits due to enhanced plasticity of synapses on parvalbumin-expressing inhibitory interneurons. Thus, increased gamma burst power may be involved in producing psychotic symptoms and cognitive dysfunction.

## 1. Introduction

Electro- and magneto-encephalography (EEG/MEG) studies have consistently demonstrated that various types of sensory-evoked and cognition-related responses tend to be decreased in individuals with schizophrenia (SZ) compared to healthy control persons (HC). Event-related potentials (ERPs) ranging from early sensory evoked components like the auditory N1 (Rosburg et al., 2008) to purely cognitive components like the P300 (e.g., Ford, 1999), generally show decreased rather than increased amplitudes when affected in SZ. Likewise, event-related oscillations typically show decreased power and/or phase locking measures (e.g., Tan et al., 2013; Thuné et al., 2016).

However, there is a growing awareness that while schizophrenia is often associated with *decreases* in evoked brain activity, this disorder is conversely linked with *increases* in spontaneous brain activity. For example, there have been many reports of increased power of low frequency oscillations [delta (1–4 Hz) and/or theta (4–8 Hz) bands] in the resting EEG of SZ (reviewed in Boutros et al., 2008; Newson and Thiagarajan, 2019). Evidence for increased spontaneous brain activity in SZ has also come from functional neuroimaging studies, which have found increased activity in SZ during task baseline periods and resting state in the hippocampus (e.g., Tregellas et al., 2014), increased global brain signal (Yang et al., 2014), and increased functional connectivity in prefrontal cortical networks (e.g., Anticevic et al., 2015) and the default mode network (e.g., Whitfield-Gabrieli et al., 2009). There is also evidence for increased cortical excitability in SZ from transcranial magnetic stimulation studies (TMS), as slow repetitive TMS reduces auditory hallucinations in treatment-intractable SZ (e.g., Hoffman and Cavus, 2002), and motor cortex inhibitory mechanisms show deficits in schizophrenia (reviewed in Daskalakis et al., 2007). Thus, there is good evidence for several kinds of increased spontaneous brain activity in schizophrenia.

There have also been reports of increased spontaneous high frequency [beta (13–30 Hz) and gamma (30–100 Hz) band] activity in SZ during the resting state, which go back decades (reviewed in Itil, 1977), but the contribution of artifacts to those reports has been debated. Only fairly recently have modern artifact-removal methods been applied to resting state EEG recordings to exclude the possibility of high-frequency artifacts (e.g., Keren et al., 2010). While some studies have found increased spontaneous gamma activity (SGA) during the resting state in SZ (e.g., Tanaka-Koshiyama et al., 2020), most have not (reviewed in Boutros et al., 2008; Newson and Thiagarajan, 2019). However, increased SGA has been reported in the auditory cortex in schizophrenia during auditory steady-state stimulation (Spencer, 2012; Hirano et al., 2015; Parker et al., 2019). This phenomenon has been correlated with auditory hallucination symptoms (Hirano et al., 2015) and could reflect the dysfunction of *N*-methyl-D-aspartate receptors (NMDARs) on parvalbumin-expressing inhibitory interneurons (PVIs), as pharmacological and genetic models of NMDAR hypofunction on PVIs frequently show increased SGA (e.g., Carlén et al., 2012; Hunt and Kasicki, 2013; Guyon et al., 2021; McNally et al., 2021). Broadband SGA is currently thought to reflect asynchronous neuronal spiking in cortical circuits (e.g., Manning et al., 2009; Whittingstall and Logothetis, 2009; Scheffer-Teixeira et al., 2013; Burke et al., 2015), and indexes synaptic excitation/inhibition (E/I) balance (Yizhar et al., 2011).

Recent studies have suggested that in typical analysis methods of oscillatory activity, in which measures are derived across trials, important information is obscured concerning the within-trial dynamics of oscillations. While activity averaged across trials may appear to be a sustained oscillation, the averaging process can hide the fact that the apparently sustained oscillation actually consists of brief bursts of oscillations that occur at different times (and possibly frequencies) across trials (Jones, 2016; Lundqvist et al., 2016; van Ede et al., 2018). To date, findings of increased SGA in SZ have come from time-averaged spectra, so it is not known whether increased SGA occurs at a constant level or rather in bursts. It is also unknown whether increased SGA is related to the slope of the 1/*f* EEG spectrum, which also indexes the E/I ratio (e.g., Miller et al., 2009; Podvalny et al., 2015; Gao et al., 2017). Thus, elucidating the dynamics underlying increased SGA in SZ would help us to better understand the alterations in neural circuit function that occur in schizophrenia. It could also give us deeper insight into the nature of cognitive dysfunction in this disorder, as there is evidence that oscillatory bursts subserve particular cognitive functions such as working memory maintenance and readout (Lundqvist et al., 2018; Miller et al., 2018).

Here we examined the contributions of gamma bursting and spectral slope to the increased broadband SGA effect during auditory steady-state stimulation in schizophrenia in a re-analysis of the SGA data first reported in Hirano et al. (2015). In that study, we analyzed SGA differences between SZ and HC in terms of auditory steady-state stimulation frequency [20/30/40 Hz; 40 Hz stimulation typically evokes a maximal auditory steady-state response (ASSR)], period of the epoch (pre-stimulus baseline vs. ASSR period, to determine if stimulus presentation affected SGA), hemisphere [left vs. right hemisphere (LH/RH), as schizophrenia is characterized by LH abnormalities], and dipole (tangential vs. radial, to account for potential differences in SGA in the dorsal vs. medial surfaces of Heschl’s gyrus). In Hirano et al. (2015) there was an overall increase in SGA in SZ compared to HC, which was pronounced for 40 Hz stimulation in the LH dipoles. SGA during 40 Hz stimulation in the LH also was correlated with auditory hallucination symptoms and gray matter volume of Heschl’s gyrus (primary auditory cortex; Hirano et al., 2020), while SGA in other conditions was not.

In the present study we hypothesized that if gamma bursts made a principal contribution to increased SGA in SZ, gamma burst power, burst count, and/or extent (area) in the time/frequency (TF) map would be larger in SZ than HC. In contrast, power in the TF map outside of the gamma bursts, and spectral slope, would not contribute to the increased SGA effect in SZ. We did not have *a priori* hypotheses about the effects of the other factors in Hirano et al. (2015) on gamma bursts.

## 2. Materials and methods

Except where noted, software was written in the IDL programming environment (Harris Geospatial Solutions, Inc.). All non-proprietary code is freely available upon request.

### 2.1. Participants

Participants in this study were 24 chronic SZ (4 female, 20 male) and 24 HC (4 female, 20 male) matched to the SZ group on age (HC: 44.1 ± 7.3 years; SZ: 46.0 ± 9.1 years; *p* = 0.439) and parental socio-economic status. Full details can be found in Hirano et al. (2015). All participants gave informed consent and were reimbursed for their participation. This study was approved by the Institutional Review Boards of the VA Boston Healthcare System and Harvard Medical School.

### 2.2. EEG recording and processing

For complete details, please see Hirano et al. (2015). Participants listened to 150 click trains for each stimulation frequency (500 ms duration, 1100 ms stimulus onset asynchrony). Click train stimulus frequencies were 20, 30, and 40 Hz. The EEG was recorded from 71 standard electrode sites with a Biosemi ActiveTwo system at 512 Hz (0.01–103 Hz passband). During recording the electrodes were referenced to the system’s internal loop (CMS/DRL electrodes). The channels were re-referenced offline to the left mastoid for subsequent processing steps. Ocular, muscle, saccadic spike potential, and electrocardiographic artifacts were identified and removed with independent component analysis (ICA) in MATLAB (Mathworks Inc.) using the script *runica.m* from the EEGLAB toolbox (Delorme and Makeig, 2004). ICs representing artifacts were identified based on their topographic, temporal, and spectral signatures (e.g., Keren et al., 2010; Shackman et al., 2010). Additional artifact criteria were: (1) > ± 90 μV change in one time point, and (2) amplitude range within an epoch exceeding 200 μV. An artifact scan was run prior to artifact IC removal, and then afterward, to exclude any residual artifacts. Artifact-free single epochs were then re-referenced to the average reference. The number of artifact-free epochs did not differ between HC (139 ± 13) and SZ (138 ± 13).

### 2.3. Source localization

In measuring SGA, it is critical to avoid contamination by high-frequency muscular and ocular artifacts. In addition to excluding these artifacts using the ICA procedure described above, source localization methods can be used to create spatial filters that focus on intracranial activity and exclude extracranial activity (Hipp and Siegel, 2013; Muthukumaraswamy, 2013). In the human brain, EEG activity in the auditory cortex typically manifests at the scalp with a maximum at fronto-central electrodes and minima of opposite polarity at lateral temporal electrodes, particularly at the mastoids (when a nosetip or average reference is used). This spatial pattern can be accounted for by pairs of equivalent current dipoles in each hemisphere in the superior temporal plane. Equivalent current dipoles represent source activity as dipoles at discrete points within the brain, based on the assumption that the sources are focal (which seems to be accurate for early sensory-evoked activity). Each dipole pair contains one dipole pointing toward the fronto-central scalp (tangential to the side of the head) and another dipole pointing toward the lateral temporal scalp (radial to the side of the head) (Sometimes a 5th dipole in a deep, medial location is included to account for subcortical activity). Dipole modeling has proven successful at localizing auditory cortex activity, including the ASSR, which localizes to primary auditory cortex (e.g., Herdman et al., 2002, 2003; Poulsen et al., 2007).

In Hirano et al. (2015) we used the localization of the ASSR to create a spatial filter for primary auditory cortex activity to better measure SGA. The grand average 40 Hz ASSR in HC (which had the highest signal-to-noise ratio) was localized using the BESA v5.1.8 package (BESA GmbH) in a 4-dipole model: 2 pairs of tangential and radial dipoles in the auditory cortex of each cerebral hemisphere. The head model was the standard BESA 4-shell (brain, scalp, skull, and cerebrospinal fluid) spherical head model. During the dipole fitting process, the tangential and radial dipoles in each hemisphere were constrained to have the same locations but free orientations. The locations of the dipole pairs in each hemisphere were constrained to be symmetric. The single trial data from each participant were then forward-projected through the dipole model to obtain source estimates of auditory cortex activity (see Hirano et al., 2015 for further details).

### 2.4. Burst analyses

**FIGURE 1:**
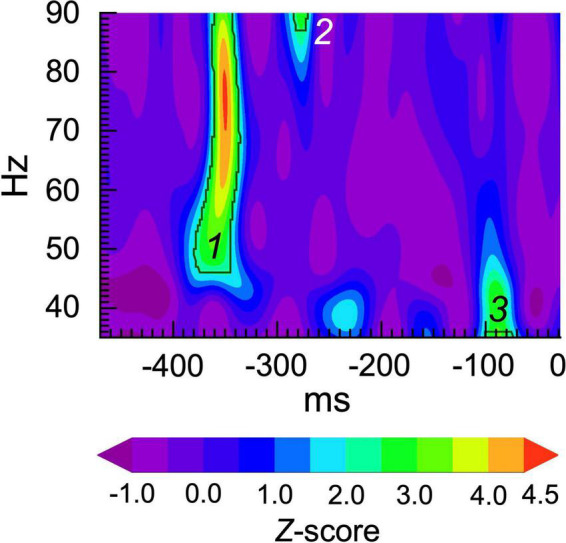
Gamma burst classification procedure. The plot shows a *Z*-score map of single trial power. The black contours indicate the threshold for *Z* = > 2.0 for at least 1 cycle at each frequency. In this trial, 3 bursts were detected at approximately: (1) –380 to –340 ms, 46-90 Hz; (2) –290 to –270 ms, 87 to 90 Hz; and (3) –100 to –70 ms, 35 to 37 Hz.

We performed spectral burst analysis following the general approach of Lundqvist et al. (2016), in which bursts at the single trial level were defined as TF windows in which spectral power exceeded a particular statistical threshold for at least 1 cycle. Each single trial time series was transformed into a TF map of spectral power using the Morlet wavelet transform (f0/σf = 6) (as implemented in IDL by Torrence and Compo, 1998), and then transforming the power values into *Z* scores The central frequencies of the wavelets ranged from 35 to 90 Hz at 1 Hz steps. TF maps of event-related spectral power were computed for each single trial. The baseline (−500 to 0 ms) and ASSR (30–530 ms) periods were scanned for bursts at each wavelet frequency. To avoid edge artifacts from the wavelet transform, the baseline and ASSR periods were shortened to −470 to −30 ms and 60 to 500 ms windows, respectively (440 ms each). Bursts were defined as consecutive time points of at least one cycle duration during which the *Z* score of power exceeded 2 standard deviations above the trial-wide average value. See Figure 1 for an example.

We extracted 3 burst parameters for each condition in each participant’s data set: (1) burst power (averaged over TF points and bursts); (2) burst count (averaged number of bursts per trial); and (3) burst area (number of TF points spanned by bursts, averaged over bursts). We also measured the power in the TF map outside of the bursts (averaged across trials), which we term here “non-burst trial power.”

### 2.5. Power spectrum analyses

Spectra for each condition were obtained by pre-multiplying each 500 ms epoch by a periodic Hann window and averaging the squared moduli of the Fourier transformed time series over non-overlapping segments using the signal processing toolkit in the Python package SciPy. We applied Barlett’s method as opposed to Welch’s overlapping tapers or the multitaper approach as inspection of representative spectra using both methods showed little improvement in smoothing and bias reduction. Moreover, this was the approach used in prior analysis of these data (Hirano et al., 2015) and we applied it here to ensure consistency among consecutive analyses.

Spectral slope analyses used the FOOOF method (Donoghue et al., 2020), which decomposes EEG power spectra into periodic (oscillatory) and aperiodic components. For each participant, we fit a separate FOOOF model to each condition. Raw power spectra were entered into the model fitting subroutines from the FOOOF Python package, where the data were converted from linear to dB units and modeled as a combination of Gaussian peaks and an exponential aperiodic roll-off. For the periodic component, we set the minimal peak height to 0.1 μV^2^/Hz with a peak bandwidth range of 4–12 Hz. We set the aperiodic fitting procedure to include a “knee” component to capture the bend in the spectra observed at ∼30 Hz, where the slope becomes less steep (see Equation 3 of Donoghue et al., 2020). The slope parameter *X* (in 1/*f*^[–^*^X]^*) returned by the model was saved for each condition. Smaller values of *X* indicate a flatter (less negative) slope of the EEG spectrum, while larger values of *X* indicate a steeper (more negative) slope.

### 2.6. Statistical analyses

Statistical analyses were performed using SPSS 29.0. The main dependent variables were analyzed in mixed-model ANOVAs with the factors Group (HC/SZ), Stimulation Frequency (20/30/40 Hz), Period (baseline/ASSR), Hemisphere [left/right hemisphere (LH/RH)], and Dipole (tangential/radial). Type I error rate was 0.05. The Greenhouse-Geisser correction for inhomogeneity of variance was applied for factors with more than 2 levels and is reflected in the reported *p*-values (Keselman and Rogan, 1980).

Multi-factor ANOVAs can present the largely unrecognized problem of inflated Type I error rate when effects in the model are analyzed without being constrained by *a priori* hypotheses or correction of *p*-values for multiple tests (Cramer et al., 2016; Luck and Gaspelin, 2017). To address this potential problem, we specified the critical tests for our hypothesis, and corrected all other significant effects for multiple tests. Our *a priori* hypothesis was that gamma bursts would make a major contribution to increased SGA in SZ through: (1) increased average burst power in SZ; (2) increased number of bursts in SZ; and/or (3) increased duration/bandwidth (area) of bursts in SZ. In contrast, we predicted that power in the TF map outside of the gamma bursts would not make a major contribution to increased SGA in SZ. Therefore, the Group effect in the ANOVAs was the main test of our hypothesis, and the *p*-values for this effect were not corrected, while for the rest of the effects the critical *p*-value was 0.00161 (with 5 factors, the number of main effects and interactions was 2^5^−1 = 31, so 0.05/31 = 0.00161; Cramer et al., 2016). The same approach was used for the spectral slope analyses. Stepwise linear regression was used to determine the contribution of the burst parameters and spectral slope to SGA.

## 3. Results

### 3.1. Burst parameters

**FIGURE 2:**
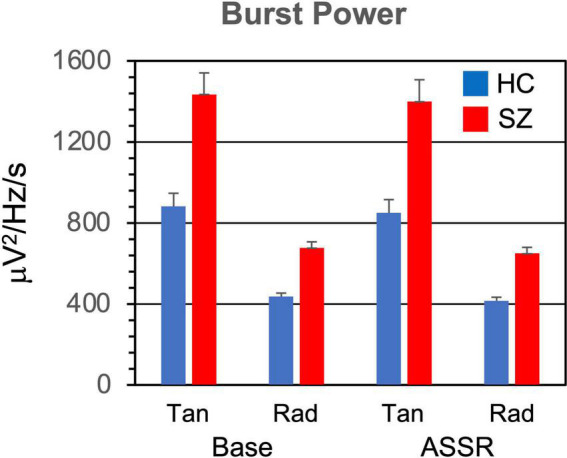
Gamma burst power (averaged across TF points and bursts) grand means for each participant group, period, and dipole. Error bars indicate standard error. HC, healthy controls; SZ, schizophrenia patients; Tan, tangential dipole; Rad, radial dipole; Base, baseline period of epoch; ASSR, ASSR period of epoch.

**FIGURE 3:**
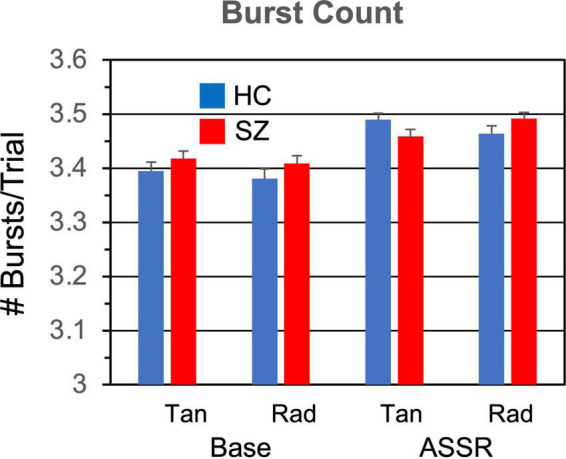
Gamma burst count (number of bursts per trial) grand means and standard errors for each participant group and condition.

**FIGURE 4:**
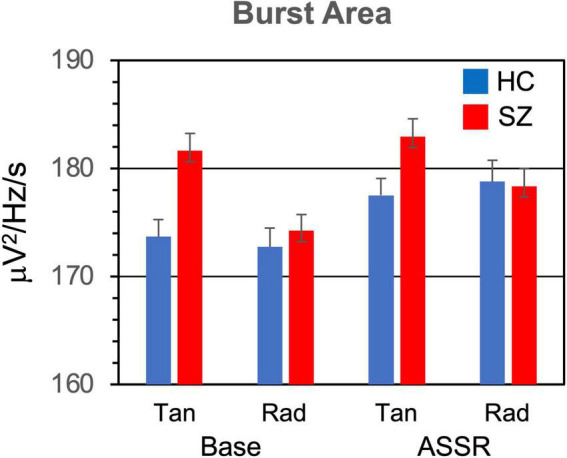
Gamma burst area (area in TF map averaged across bursts) grand means and standard errors for each participant group and condition.

**FIGURE 5:**
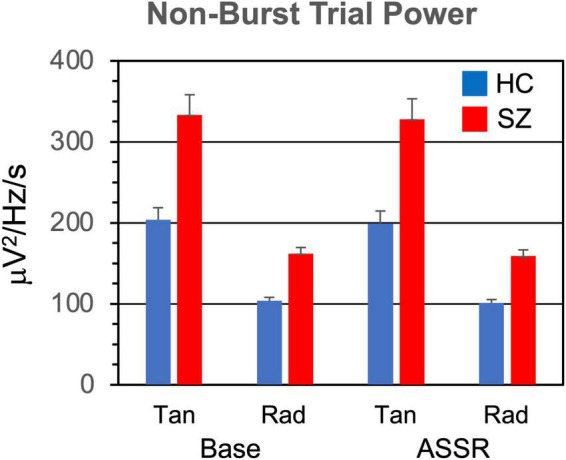
Non-burst trial power (power in TF map outside of bursts, averaged across TF points, and trials) grand means and standard errors.

The means and standard errors of the burst parameters and non-burst trial power are presented in Figures 2–5.

#### 3.1.1. Burst power

Power within the gamma bursts (Figure 2) was higher in SZ than HC [*F*_(1,46)_ = 7.69, *p* < 0.01]. Burst power was also higher during the baseline than the ASSR period [*F*_(1,46)_ = 31.4, *p* < 0.001], and higher in the tangential dipoles than the radial dipoles [*F*_(1,46)_ = 31.4, *p* < 0.001].

#### 3.1.2. Burst count

The number of gamma bursts per trial (Figure 3) did not differ between SZ and HC [*F*_(1,46)_ = 0.119, *p* = 0.731], but there were more gamma bursts in the ASSR period than the baseline period [*F*_(1,46)_ = 54.5, *p* < 0.0001].

#### 3.1.3. Burst area

The average area of gamma bursts in the TF map (Figure 4) did not differ between SZ and HC [*F*_(1,46)_ = 0.776, *p* = 0.383]. Burst area was larger for bursts in the ASSR period compared to the baseline period [*F*_(1,46)_ = 28.5, *p* < 0.001].

The Stimulation Frequency X Period interaction was significant [*F*_(2,92)_ = 9.16, *p* < 0.001]. In decomposing this interaction, we further adjusted the critical *p*-value, dividing by 2 to yield 0.000805. There was a significant effect of Stimulation Frequency on burst area in the ASSR period [*F*_(2,92)_ = 10.3, *p* < 0.001], but not in the baseline period [*F*_(2,92)_ = 1.52, *p* = 0.226]. Decomposition of the Stimulation Frequency effect in the ASSR period (with further *p* correction) did not yield significant comparisons.

The Period X Dipole interaction was also significant [*F*_(1,46)_ = 13.5, *p* < 0.001]. Decomposition of this interaction (with a critical *p* of 0.000805) revealed a significant effect of Period (baseline < ASSR) for the radial dipoles [*F*_(1,46)_ = 44.7, *p* < 0.0001] but not the tangential dipoles [*F*_(1,46)_ = 9.65, *p* = 0.00324].

### 3.2. Non-burst trial power

Power in the TF maps outside the gamma bursts (Figure 5) was higher in SZ than HC [*F*_(1,46)_ = 7.81, *p* < 0.01]. Non-burst power was also higher during the baseline period than the ASSR period [*F*_(1,46)_ = 12.7, *p* < 0.001], and higher in the tangential than the radial dipoles [*F*_(1,46)_ = 35.8, *p* < 0.001].

### 3.3. Spectral slope

**FIGURE 6:**
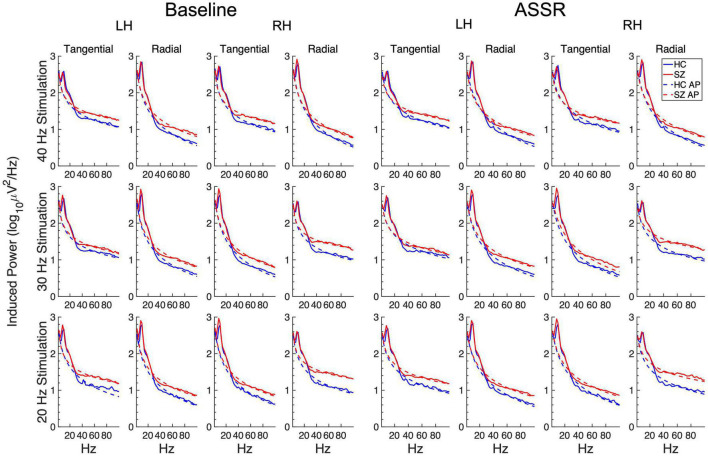
Grand average induced power spectra for each participant group and condition. The original spectra are plotted in solid lines, and the aperiodic portions (AP) of the spectra (estimated from the FOOOF algorithm) are plotted in dashed lines.

**FIGURE 7:**
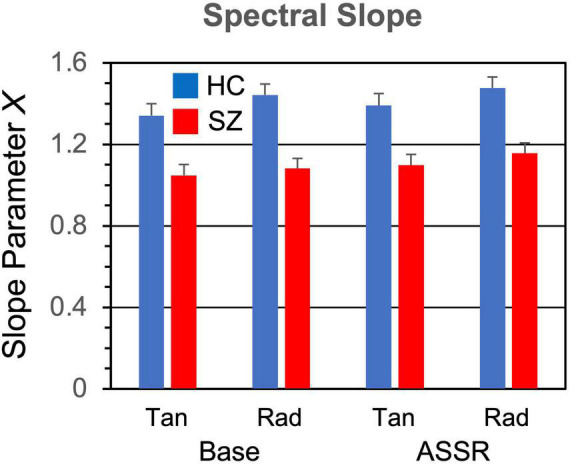
Spectral slope parameter (1/*f*^[^*^–X^*^]^) grand means and standard errors.

The induced power spectra in the baseline and ASSR periods are shown in Figure 6. The original spectra are overplotted with the aperiodic spectra estimated by the FOOOF algorithm, and the slope means are given in Figure 7. The slope parameter extracted by the FOOOF algorithm differed significantly between HC and SZ [*F*_(1,46)_ = 5.77, *p* < 0.05], with SZ having a smaller exponent than HC, indicating that SZ had a less negative (flatter) spectral slope than HC. Spectral slope was also lower (flatter) in the baseline period than the ASSR period [*F*_(1,46)_ = 34.4, *p* < 0.001].

Three interaction effects were also significant. Frequency X Dipole [*F*_(2,92)_ = 36.0, *p* < 0.001] did not yield further significant effects when decomposed (critical *p* = 0.000805). The Hemisphere X Dipole interaction [*F*_(1,46)_ = 61.4, *p* < 0.001] resulted from opposite patterns of hemispheric laterality for the tangential [RH > LH; *F*_(1,46)_ = 53.7, *p* < 0.0001] and radial dipoles [LH > RH; *F*_(1,46)_ = 38.8, *p* < 0.0001]. Lastly, the Frequency X Hemisphere X Dipole interaction [*F*_(2,92)_ = 29.6, *p* < 0.001] reflected significant Frequency X Hemisphere interactions for the tangential [*F*_(2,92)_ = 9.83, *p* < 0.001] and radial [*F*_(2,92)_ = 40.4, *p* < 0.0001] dipoles. Further decomposition of these interactions (critical *p* = 0.000403) revealed a significant effect of Frequency [*F*_(2,92)_ = 17.8, *p* < 0.0001] for the RH radial dipoles (40 Hz > 30 Hz, *p* critical = 0.000134, *p* < 0.001).

### 3.4. Regression analysis of SGA

We used stepwise linear regression modeling to test the degrees to which the burst parameters, non-burst trial power, and spectral slope made contributions to SGA. The dependent variable was spontaneous gamma power averaged across ASSR stimulation frequencies, baseline and ASSR periods, hemispheres, and dipoles. The predictors were Group, burst power, burst count, burst area, non-burst trial power, and spectral slope (all averaged over the above conditions). This procedure yielded 3 significant models. In the first model [*F*_(1,46)_ = 450, *p* < 0.001, *R*^2^ = 0.905], burst power (β = 0.953) was the only predictor selected. In the second model [*F*_(2,45)_ = 250, *p* < 0.001, *R*^2^ = 0.917], spectral slope (β = −0.144) was added to burst power (β = 0.849). And in the third model [*F*_(3,44)_ = 180, *p* < 0.001, *R*^2^ = 0.925], Group (β = −0.093) was added to burst power (β = 0.875) and spectral slope (β = −0.156).

**FIGURE 8:**
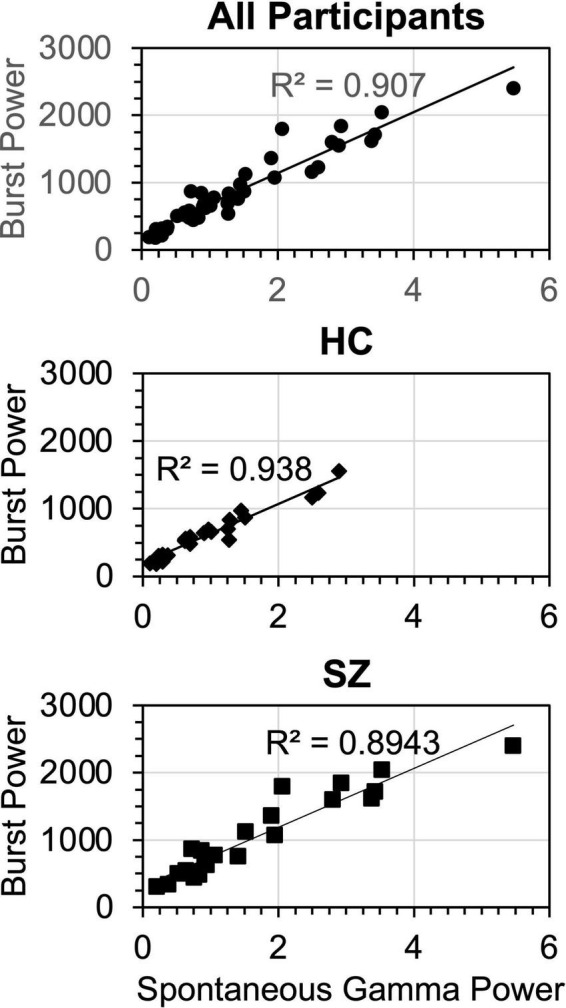
Regression functions for gamma burst power vs. spontaneous gamma power, plotted for the regression models fitted across all participants, HC only, and SZ only. Units for burst power (averaged over TF points and bursts) were μV^2^/Hz/s. Units for spontaneous gamma power were μV^2^/Hz (Burst power was calculated from the wavelet transform and spontaneous gamma power was calculated from the Fast Fourier Transform).

Regression analysis was repeated on the HC and SZ data separately. For HC, one model was found [*F*_(1,23)_ = 331, *p* < 0.001, *R*^2^ = 0.938], in which the sole predictor was burst power (β = 0.968). Similarly, for SZ one model was found [*F*_(1,23)_ = 187, *p* < 0.001, *R*^2^ = 0.895], in which the only predictor was burst power (β = 0.946). The gamma burst power regression plots are shown in Figure 8.

These analyses show that gamma burst power was by far the main contributor to SGA, accounting for over > = 90% of the variance in the participant groups. Spectral slope and Group accounted for less than 3% of the variance in the overall regression. Non-burst trial power, while being significantly increased in SZ compared to HC, did not make a significant contribution to spontaneous gamma power despite occupying the same frequency band.

## 4. Discussion

The results of this study suggest that SGA may consist both of bursts of gamma activity at irregular intervals and frequencies, plus a more constant level of background activity that is represented by non-trial burst power and possibly spectral slope. These findings support our hypothesis that gamma bursts, rather than a sustained degree of gamma power or a less negative 1/*f*^(–^*^X^*^)^ spectral slope, make the most important contribution to the increased SGA seen in the auditory cortex in schizophrenia during auditory steady-state stimulation. Gamma bursts in SZ had more power than in HC, while the number of gamma bursts or the TF extent of the bursts did not differ between groups. While non-burst gamma power was higher and spectral slope was less negative in SZ than HC, regression modeling indicated that by far, the largest contributor of these measures to SGA was gamma burst power.

In addition, we found that SGA and gamma bursting were affected by stimulus processing. Burst power and non-burst trial power were larger in the baseline period than the ASSR period, and spectral slope was less negative (flatter). In contrast, burst count and burst area were larger during the ASSR period than the baseline period. Together, these results suggest that SGA overall was possibly suppressed during the ASSR relative to the baseline period, although there were more bursts with a greater TF extent during the ASSR period. The effects of stimulus processing on SGA need to be studied in more detail.

Concerning the other factors, both burst power and non-burst power were larger for tangential than radial dipoles. These effects likely reflect the generally greater power in tangential than radial dipoles for the ASSR (e.g., Herdman et al., 2002; Spencer et al., 2009; Hirano et al., 2015), as the radial dipoles are likely located in the medial part of Heschl’s gyrus, deep within the brain (for example see Figure 1C in Hirano et al., 2020). There were no major effects of interest involving the factors Stimulation Frequency and Hemisphere.

Spontaneous gamma activity is currently thought to reflect asynchronous spiking activity in the cortex (Manning et al., 2009; Whittingstall and Logothetis, 2009; Scheffer-Teixeira et al., 2013; Burke et al., 2015). However, analysis of the within-trial dynamics underlying SGA suggests that it consists of short bursts of gamma oscillations that occur at irregular intervals (Lundqvist et al., 2016, 2018), and SGA may reflect dendritic as well as spiking activity in distinct cortical layers (Leszczyński et al., 2020). To date, little research has been done on the precise roles that gamma bursts may play in information processing in the brain. Lundqvist et al. (2016) found that bursts of gamma oscillations in the prefrontal cortex of monkeys were involved in encoding items into WM and initiating the maintenance of those items via rapid processes that they proposed involved short-term synaptic plasticity. Gamma bursts were also involved in the retrieval of items from WM (Lundqvist et al., 2018). These roles are consistent with a computational modeling study which found that gamma bursts could be an effective mechanism for transiently synchronizing cortical circuits in different brain regions to route information flow between them (Palmigiano et al., 2017). To elucidate the functional significance of gamma bursts during working memory, additional studies are necessary to test these hypotheses, for example by determining whether gamma bursts occur specifically for cell assemblies that code remembered items, and manipulating the activity of these assemblies to affect task performance. Furthermore, the work of Palmigiano et al. would predict that gamma bursts should be synchronized between task-relevant brain regions, which can be tested in multi-areal recordings (e.g., prefrontal and sensory areas).

While there have been abundant reports of increased SGA in animal models of NMDAR hypofunction (reviewed in Carlén et al., 2012; Hunt and Kasicki, 2013; Guyon et al., 2021; McNally et al., 2021), the dynamics underlying SGA have not been examined in these models. Some studies of NMDAR hypofunction animal models have found altered burst patterns in pyramidal cells which could contribute to increased SGA, such as increased burst duration (Carlén et al., 2012) and increased size of calcium transients (putative action potential bursts in 2-photon imaging; Hamm et al., 2017; Seshadri et al., 2018), but also reduced bursting (Jackson et al., 2004). These results have been inconsistent, likely due to differences in recording conditions (e.g., *in vitro* preparations, *in vivo* anesthetized/restrained conditions, freely behaving) and sampling of cells (e.g., Kargieman et al., 2007).

We propose that increased gamma burst power results from PVI dysfunction due to hypofunction of NMDARs on these neurons. Perineuronal nets surrounding PVIs are degraded in SZ in the prefrontal cortex (Enwright et al., 2016) and the auditory system (Kilonzo et al., 2020), and perineuronal net reduction returns PVIs to a state of juvenile-like enhanced plasticity that is associated with increased excitation, enhanced SGA (Lensjø et al., 2017), and decreased evoked gamma power (Carceller et al., 2020). Increased gamma burst power in SZ could be associated with improper strengthening of synaptic connections between neural circuits due to increased PVI plasticity, which in turn could disrupt cognitive processes like WM maintenance and retrieval. In support of this hypothesis, McNally et al. (2021) found that optogenetic stimulation of basal forebrain PVIs in mice increased frontal SGA and disrupted WM performance. Gamma bursts could play a similar role in generating psychotic symptoms by making attractor states in neural circuits less stable and/or encouraging the unconstrained formation of new cell assemblies (Rolls et al., 2008). Consistent with this idea, ketamine administration produces schizophrenia-like cognitive and perceptual alterations in healthy persons (Krystal et al., 2003), increases plasticity in cortical circuits (e.g., Cornwell et al., 2012; Duman and Aghajanian, 2012), and increases SGA (e.g., Hong et al., 2010; Muthukumaraswamy et al., 2015; Rivolta et al., 2015).

One test of our hypothesis would be to determine if the increased SGA resulting from NMDAR antagonists reflects increased gamma burst power and not effects on other components of the EEG spectrum. Furthermore, as this effect would be dependent upon the integrity of perineuronal nets surrounding PVIs, the degradation of these nets (in animal models) should lead to increased gamma burst power. In humans, the relationship between gamma bursting and working memory processes needs to be studied, particularly how gamma burst power may be related to working memory deficits in schizophrenia.

In summary, in this paper we present evidence that increased SGA in the auditory cortex of SZ is due to increased power of gamma bursts, which seems to be independent of other measures of spontaneous neural activity, such as spectral slope and non-burst trial power. These dissociations in relation to SGA suggest that these measures reflect different aspects of spontaneous activity that might make separate contributions to the EEG spectrum. Further research will be necessary to determine if these measures reflect different network mechanisms, and to relate them more closely to the psychiatric and cognitive disturbances associated with schizophrenia.

## Data availability statement

The original contributions presented in this study are included in the article/supplementary material, further inquiries can be directed to the corresponding author.

## Ethics statement

The studies involving human participants were reviewed and approved by the VA Boston Healthcare System IRB, Harvard Medical School ORSP. The patients/participants provided their written informed consent to participate in this study.

## Author contributions

KS conceived the study, performed burst analyses and statistical analyses, and wrote the manuscript. AN performed burst and aperiodic spectral analyses and contributed to the manuscript. YH processed and analyzed the original EEG data including source localization, spectral analyses, and statistical analyses. ML helped to conceive this study. All authors approved the manuscript.
